# Characterization of Lung Microbiome in Subclinical Pneumonic Thai Pigs Using 16S rRNA Gene Sequencing

**DOI:** 10.3390/ani15030410

**Published:** 2025-02-02

**Authors:** Phacharaporn Tadee, Pakasinee Khaodang, Prapas Patchanee, Songphon Buddhasiri, Thanaporn Eiamsam-ang, Nattinee Kittiwan, Pakpoom Tadee

**Affiliations:** 1 Faculty of Animal Science and Technology, Maejo University, Chiang Mai 50290, Thailand; phacharaporn.boonkhot@gmail.com (P.T.);; 2 Faculty of Veterinary Medicine, Chiang Mai University, Chiang Mai 50100, Thailand; 3 Bacteriology Section, Veterinary Research and Development Center (Upper Northern Region), Lampang 52190, Thailand

**Keywords:** pig, pig production, lung lesion, lung microbiome, 16S rRNA sequencing, Thailand

## Abstract

Bacterial respiratory disease is one of the major concerns in the modern pig industry. To address the limitations of culture-based methods, 16S rRNA sequencing was employed to characterize the pig lung microbiome to gain a better understanding of microbial physiology and their population genetics. A batch of 510 slaughtered pigs from a farm located in Lampang province, Thailand, was selected. Individual pig weight was recorded. A total of 24 lungs (10 normal and 14 pneumonic lungs) were sampled for gross lesion examination and lung microbial communities were investigated. Poor growth performance and weight uniformity were denoted in this batch. Several pathogenic bacteria were detected in both normal and pneumonic lungs. Microbial diversity was decreased in the pneumonic group. PCoA and NMDS analysis showed a clear separation between the groups. *Stenotrophomonas* spp. (42.12%) was the dominant genus identified in normal lungs, while *Mycoplasma hyopneumoniae* (71.97%) was the most abundant in pneumonic lungs, correlating with the commonly observed consolidation lesions. The slaughterhouse serves as a key checkpoint for gathering comprehensive information on pig respiratory health, and lung is representative of the lower respiratory tract for microbiomics. Monitoring of lung lesions should be implemented routinely to gain a better understanding of regional pig respiratory health.

## Simple Summary

This study represents the first comprehensive investigation of pig lung microbiome in Thailand. We employed 16S rRNA sequencing to characterize the microbial communities in the pig lungs. In the study, pathogenic bacteria were identified in both normal and pneumonic lungs, indicating that nearly all pigs in the herd are at risk of developing respiratory diseases, with some potentially acting as asymptomatic carriers. To enhance production efficiency in subsequent batches, additional measures such as alternative vaccination programs, optimized medication strategies, and stringent biosecurity protocols should be implemented.

## 1. Introduction

Respiratory disease is one of the most important health concerns in the modern pig industry worldwide [1,2,3]. Bacterial infection is a major contributing factor and is often overlooked due to its association with subclinical conditions [4,5]. Poor growth performance and increasing of secondary infection susceptibility to many pathogens are dependent on the circumstance [6]. Gross evaluation of pig lungs at the slaughter line has great potential for assessing respiratory health, detecting subclinical infections, and quantifying their impact [3,7]. However, clarifying the presence of pathogens is challenging due to the complexity of cultivation methods, especially for various fastidious organisms such as *Mycoplasma* spp., *Haemophilus* spp., and *Actinobacillus* spp. [8,9]. These organisms require specific nutrients, conditions, and extended time for growth. Additionally, only covering a small fraction of all organisms is the limitation of culture methods. It is estimated that “only 1% of prokaryotes can be cultured” [10]. Therefore, controlling pig respiratory diseases would greatly benefit from capturing entire lung communities and interpreting their relationship with lung lesions [11].

To characterize bacterial communities or microbiomes in pig’s lung, the high-throughput sequencing of the 16S ribosomal RNA (rRNA) gene, a small and highly conserved locus, has been utilized for bacterial taxonomic profiling. This approach overcomes the challenge of culturing microbial species that are difficult to culture [12,13,14]. Early 16S rRNA sequencing studies in animals mostly focused on the gut microbiota, possibly as it is minimally invasive to collect stool samples, and because the higher yield of microbial material in the samples make it easier to extract microbial DNA [15]. As DNA extraction techniques are constantly improving, researchers have become better equipped at profiling from the respiratory system [15,16]. Dickson et al., 2016 [17] stated that “The lungs are constantly exposed to diverse communities of microbes from the oropharynx and other sources”. In field practices, there are fewer opportunities to collect lung samples from healthy companion animals. However, this limitation is mitigated when studying livestock raised in herds, such as pigs, as samples can be obtained during the routine slaughtering process [11].

Our present study focused on the pig lung microbiome using 16S rRNA sequencing and its relationship with the lung lesion. Regarding this, the knowledge will enable a better understanding of microbial physiology, their population genetics, and the community ecology that is linked with pig health. In veterinary practice, understanding the pig respiratory microbiome and its relationships with the host organism may be useful in disease control and growth performance improvement. From a public health perspective, the discovery of various biomolecules contaminating the pig respiratory tract could raise awareness about the introduction of non-native species and the potential impacts on human health.

## 2. Materials and Methods

### 2.1. Ethical Statement

All procedures involving experimental pigs were conducted under ethical approval reference number R23/2567 from the Animal Care and Use Committee of the Faculty of Veterinary Medicine, Chiang Mai University (FVM—ACUC).

### 2.2. Experimental Pigs and Sampling Procedures

**Figure 1:**
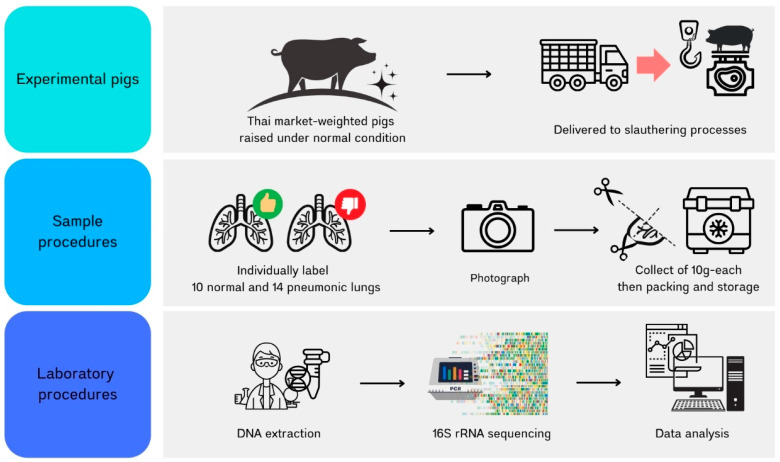
A schematic diagram of the entire workflow for this study.

A schematic diagram of the entire workflow for this study is summarized in Figure 1. The study focused on market-weight, crossbred pigs (½ Duroc × ¼ Large White × ¼ Landrace) supplied at a commercial slaughterhouse in Chiang Mai, Thailand. A batch of 510 pigs (from a herd of 693 pigs) raised at a commercial nursery-to-finishing farm under normal evaporative housing conditions in Soem Ngam district, Lampang province, Thailand (18°03′48″ N, 99°14′57″ E) was chosen for our study. These pigs were supplied from a breeder farm at around 2 weeks of age. They were fed commercial pellet feed according to the standard nutritional requirements with ad libitum water. All these pigs were immunized with three vaccines, including for porcine circovirus 2 + classical swine fever, and foot and mouth disease, at 5 and 7 weeks of age, respectively.

After overnight fasting, all 510 pigs aged approximately 23 weeks were weighed individually before slaughter. At the evisceration step, the esophagus, trachea, and lungs were removed from each carcass. Lung observations were performed, with 10 normal lungs and 14 pneumonic lungs selected. The entirety of the lungs were photographed. For each lung sample, 10 g of lung tissue from seven lobes was collected, transferred into plastic bags, and frozen on dry ice for laboratory processing within 8 h at the Faculty of Veterinary Medicine, Chiang Mai University. The samples were cut into small pieces, homogenized, and then stored at −20 °C until DNA extraction.

### 2.3. DNA Extraction and 16S rRNA Gene Sequencing

The frozen lungs were finely chopped to ensure thorough and consistent tissue disruption. Fifty mg of the homogenized material was then taken for DNA extraction using the High Pure PCR Template Preparation Kit (Roche, Basel, Switzerland) according to the manufacturer’s instructions. Following DNA isolation, the concentrations were measured using a Qubit fluorometer (Thermo Fisher Scientific, Waltham, MA, USA). The quality of DNA samples was evaluated using 2% agarose gel electrophoresis. The V3-V4 region of the 16S rRNA gene was amplified using V3-V4 338F/806R 16S rRNA primers. The libraries were sequenced on an Illumina paired-end platform (Illumina, San Diego, CA, USA) to generate 250 bp paired-end raw reads, according to the manufacturer’s instructions. All PCR amplification and sequencing steps were carried out at Novogene Co., Ltd. (Beijing, China).

### 2.4. Data Analysis

The short reads data were imported into QIIME2 v.2021.8 [18]. To clean the raw data obtained from sequencing, DADA2 was used for splicing, filtering, and noise reduction, ensuring more accurate and reliable results for bioinformatic analysis [19]. After that, the reads were merged, and taxonomy was assigned by matching Amplicon Sequence Variants (ASVs). In QIIME2, the representative sequence of each ASV was annotated to obtain the corresponding taxa at 97% similarity, using the SILVA reference database [20] to acquire the feature table. Abundance at the levels of kingdom, phylum, class, order, family, genus, and species was determined. The R program was used to process the visualization for all analyses. Taxa richness and evenness, as well as common and unique ASVs among the groups of lung phenotypes, were displayed. Alpha diversity was determined as all three indexes (Chao1, Shannon, and Simpson). Beta diversity was measured with principal coordinate analysis (PCoA) and non-metric multidimensional scaling analysis (NMDS) using Bray–Curtis dissimilarity to picture differences in community structure between normal and pneumonic lungs. Significant differences in taxa composition and community structure among the groups were determined using the *t*-test statistical method. Finally, amplicon annotation results were linked to corresponding functional databases, and PICRUSt2 [21] was utilized to predict and analyze their metabolic pathways in all lung samples.

## 3. Results

### 3.1. Experimental Pigs

**Figure 2:**
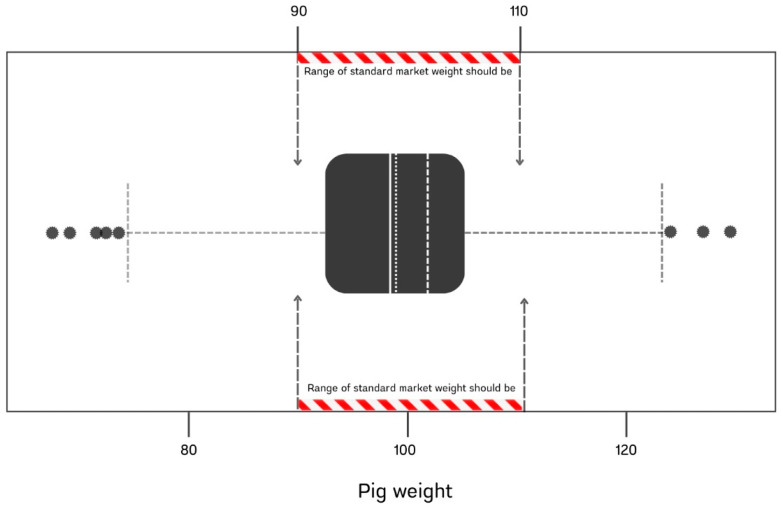
The distribution of the slaughtered weight of pigs in the studied batch (*n* = 510). Note: within the box, the vertical solid line, dotted line, and dashed line represent the median, mean, and mode values, respectively.

Among the pigs observed in the lairage area before slaughter, almost all appeared healthy. They had clear, bright eyes and responded excitedly when staff approached. Approximately 3–5% of them showed typical clinical signs of respiratory problems such as non-productive cough and mild conjunctivitis. Data of individual weights were recorded. Of 510 slaughtered pigs included in this batch, their weights ranged from 67.5 to 129.5 kg, with a 9.94 kg standard deviation. The distribution of the data was slightly negatively skewed, with a mean, median, and mode of 98.6, 99.0, and 102.0 kg, respectively (Figure 2). Additionally, five lower outliers and three upper outliers were observed. Average daily growth (ADG) was calculated, starting from 6 kg of farm entry weight until the slaughtered weight after a period of 145 days of being raised. The ADG for this batch was 0.64 kg/day. Feed conversion rate (FCR), another index that refers to animal growth efficacy, was calculated by dividing the amount of feed used with the weight gain. A total of 163,850 kg of feed was provided for 725 pigs initially, with an end number of 693 pigs. Thus, the FCR of the herd was 2.56.

### 3.2. Lung Phenotype

**Figure 3:**
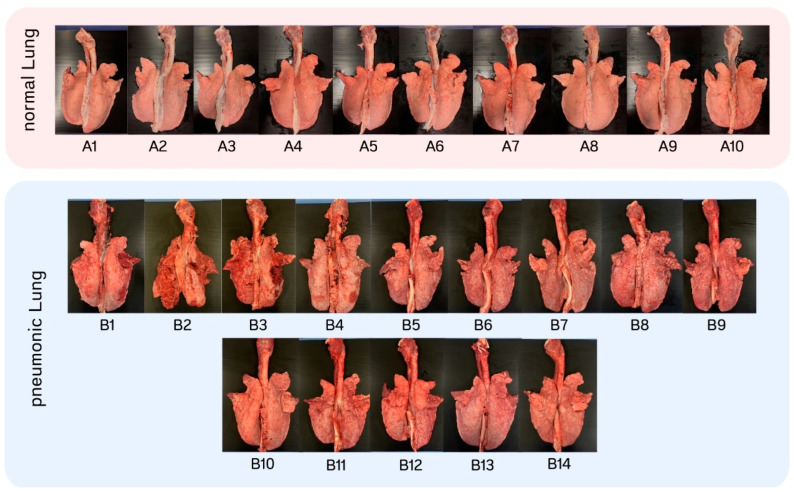
Gross appearance of 24 tested lungs.

Of 24 lung samples chosen, fourteen were grouped in pneumonic lung and ten were demonstrated as normal (Figure 3). Phenotypic patterns of pneumonia such as pleuritis, consolidation, hemorrhage, mottling, and fibrino-necrosis were noticed. Lungs which exhibited at least 10% of lesion-affected pulmonary parenchyma were grouped as pneumonic lungs. For the group of normal lungs, there were only three lungs that had no lesions in the slaughtered batch. Hence, we classified the lungs with up to 2% lesion of the parenchyma in the normal group. Description of the lesions for all lungs tested are detailed in Table 1.

### 3.3. 16S rRNA Sequencing

**Figure 4:**
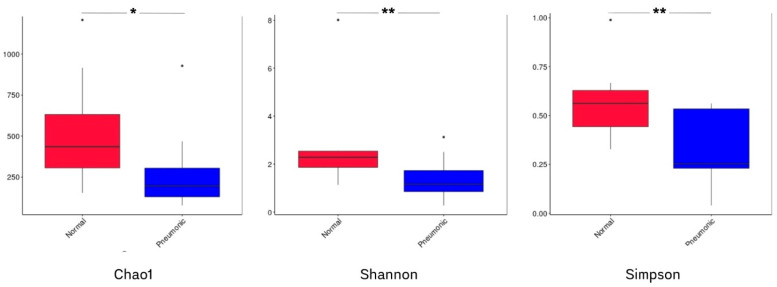
Comparative alpha diversity (Chao1, Shannon, and Simpson) of ASVs among normal and pneumonic lungs. Note: significant differences were tested using Wilcoxon *t*-test (* and ** denote 0.01 ≤ *p*-value ≤ 0.05 and *p*-value ≤ 0.01, respectively).

**Figure 5:**
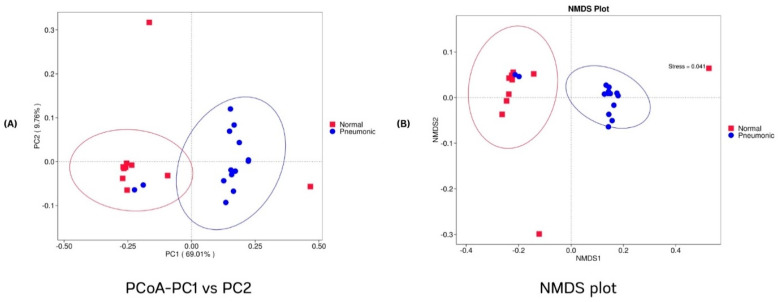
Comparative beta diversity among normal and pneumonic lungs. Note: (**A**) principal coordinate analysis (PCoA) on the ASVs based on Bray–Curtis. (**B**) Non-metric multidimensional scaling analysis (NMDS) on the ASVs based on Bray–Curtis.

A total of 24 samples were used to assess the microbiomes. After filtering for size and quality, 3,847,770 reads were obtained, with a median of 167,233 reads (minimum: 91,591; maximum: 245,330). Overall, 4478 unique bacterial amplicon sequence variants (ASVs) were identified, with 860 ASVs shared between the groups of normal and pneumonic lungs. In the comparison of alpha diversity, the Wilcoxon *t*-test indicated that all three indexes (Chao1, Shannon, and Simpson) were significantly lower in the pneumonic group (*p* = 0.02, 0.007, and 0.007, respectively) (Figure 4). To account for variations in lung microbial community composition among groups, PCoA and NMD analysis based on Bray–Curtis distances demonstrated a quite clear separation between normal and pneumonic lungs. However, four samples (A7, A8, B3, and B9) did not cluster with their groups (Figure 5A,B).

**Figure 6:**
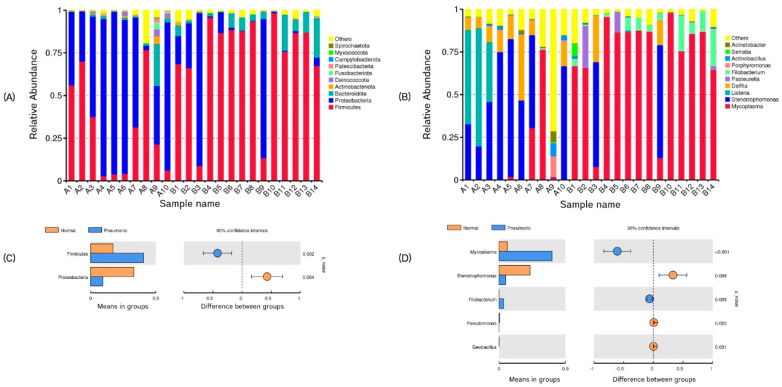
Composition of lung microbial community. Note: (**A**,**B**) relative abundance of top 10 phyla and genera, respectively (normal lung, sample A1–A10; pneumonic lung, sample B1–B14), and (**C**,**D**) significant difference of average relative abundance between normal and pneumonic lung groups in phyla and genera, respectively, using Welch’s *t*-test (normal lung, sample A1–A10; pneumonic lung, sample B1–B14).

An overview of the bacterial community profiles from 24 pig lung samples showed that the phyla *Firmicutes* (55.62%), *Proteobacteria* (34.69%), and *Bacteroidota* (5.51%) were the most predominant. Among the genera, *Mycoplasma* (46.63%) was the most dominant, followed by *Stenotrophomonas* (22.87%) and *Listeria* (6.70%). The composition of the top 10 taxa across samples is visualized in Figure 6A,B. Significant differences were observed in the average relative abundance of the phyla *Firmicutes* (31.01% for normal and 73.20% for pneumonic lungs) and *Proteobacteria* (59.69% for normal and 16.83% for pneumonic lungs) between the groups. Additionally, five genera (*Mycoplasma*, *Stenotrophomonas*, *Filobacterium*, *Pseudomonas,* and *Geobacillus*) differed significantly among the groups. Notably, *Mycoplasma* was predominantly detected in pneumonic lungs (11.14% for normal and 71.97% for pneumonic lungs), while *Stenotrophomonas* was more abundant in the normal lungs (42.12% for normal and 9.12% for pneumonic lungs) (Figure 6C,D). At the species level, *Mycoplasma hyopneumoniae* (46.62%) was the most dominant in both normal (11.14%) and pneumonic lungs (71.96%).

**Figure 7:**
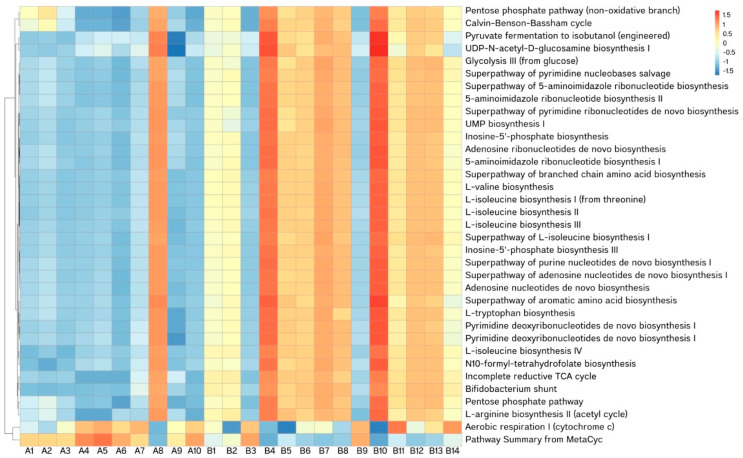
Functional analysis in bacterial genus for predictive metabolic pathways. Note: The X-axis represents the sample name, and the Y-axis represents the pathway annotation. The cluster tree on the left side of the figure is the pathway cluster tree; the corresponding value of the heatmap is the Z value of pathway relative abundance after standardization (normal lung, sample A1–A10; pneumonic lung, sample B1–B14).

Metabolic pathways of bacterial communities were predicted. The top 35 and their abundance in each sample were selected to draw the heatmap, and clustered at different levels, based on standardization to a Z value (Figure 7). Most of the samples grouped in the pneumonic lung group show a higher value than those for normal lungs, except for the samples A8, B3, and B9.

## 4. Discussion

For the slaughter batch conducted in this study, the live pigs mostly appeared healthy. Nevertheless, their weights showed poor uniformity, with nearly one-third of them (150 out of 510) deviating from the Thai standard market weight (range of 90–110 kg). Under this condition, the lighter body weight means a lower marginal benefit [22], while a higher weight means an increase in wasteful productive costs and excessive fat deposition [23]. Considering the growth rate and feed efficiency, poor performance was detected due to the lower ADG and higher FCR compared to baseline references [22]. However, the mortality rate for this herd remained within the normal range. The findings indicate that the possibility of any low–moderate virulence with highly contagious infection might be evidenced, and subclinical infection is proven [24]. This relates to the phenotypic finding of lungs observed; almost all lungs included in the batch had lesions. Since the lungs were defined as “normal” in this study, small areas of consolidation were noted. Maes et al., 2023 [3] mentioned that the extent of lung lesions is negatively correlated with growth performance, with ADG decreasing by 1.8 g/day for every 1% of lung parenchyma affected by consolidation. This is directly associated with lower slaughter weights and prolonged finishing periods. Moreover, lung lesions also play a role in meat quality by impairing muscle synthesis, which is causing changes in pH, water holding capacity, and leads to a higher risk of pale, soft, exudative (PSE) traits [25].

Due to the limitations of culture-based techniques, the belief that the healthy lung is a sterile organ has persisted for more than a decade, with the presence of microbiomes only being associated with disease states [12]. This belief is why the Human Microbiome Project, launched in 2008, did not include lung microbiome data [26]. Currently, since high-throughput next-generation sequencing methods are regularly applied in microbiology, the sequencing of the hypervariable regions of any amplicon and shotgun sequencing of the total DNA can prove that microbiomes also exist in healthy lungs [11,12,15,17,27]. In our study, we conducted the first comprehensive investigation of pig lung microbiomics in Thailand. Numerous pathogenic bacteria were detected in all samples, highlighting the complex nature of respiratory disease. High contagion rates and the co-occurrence of multiple agents are common in pigs reared under confined conditions in large communities [28]. We identified *Firmicutes* (55.62%), *Proteobacteria* (34.69%), and *Bacteroidota* (5.51%) as the most predominant phyla, showing a similar distribution to previous reports in pigs [11,27], humans [29,30], cattle [31], and mice [32]. However, the relative abundance of these three phyla fluctuates substantially with disease occurrence.

The samples grouped in the pneumonic lung category exhibited lower alpha diversity of lung microbiota compared to the normal group. A diverse microbiota can provide protection against infections; thus, a loss of diversity indicates a shift in microbiota composition which allows virulent members to proliferate more easily [16,17,27,31]. PCoA and NMDS analyses revealed that the microbiota of normal lungs differed from that of pneumonic lungs. Despite all pigs in the herd sharing the same environment, the lung microbiome of healthy pigs can maintain a dynamic equilibrium between the elimination (cough, mucociliary clearance, and immune activity) and influx of microorganisms (air inhalation and micro-aspiration from upper respiratory tract). However, when this balance is disrupted, the onset of pneumonia can occur [27,33].

Lesions observed in 24 lungs tested were of varying degrees of severity. The most common finding is that the consolidation affected the lung parenchyma. As expected, consolidation lesions are commonly associated with *Mycoplasma hyopneumoniae*, causative of enzootic pneumoniae [5,7,34]. This pathogen is confirmed as the species with the highest relative abundance found from our results. However, similar gross findings may be also observed with other bacteria such as *Pasteurella* (24.57% and 11.99% relative abundance detected from sample B2 and B5, respectively). On the other hand, *Mycoplasma hyorhinis*, *Mycoplasma hyosynoviae, Bordetella bronchiseptica*, and *Glaesseserella parasuis* as well as *Streptococcus* and *Staphylococcus* species, can be detected in consolidation lesions [3], but they were not predominant in our study. The pleuritis lesion was also common and is often strongly associated with *Actinobacillus pleuropneumoniae* [4,35]. Moreover, the pathogen is a major cause of hemorrhagic necrotizing pleuropneumonia. Interestingly, in severe pleuritis cases (sample B1–B3), the relative abundance of *Actinobacillus pleuropneumoniae* was very low (1.5%, 0.6%, and 0.2% were detected from samples B1–B3, respectively). In contrast, a higher abundance (7.6%) was detected in a normal lung sample (A9). Since pigs were not monitored over time and we examined only once, some lesions that occurred previously may have healed by the time of slaughter [3]. Furthermore, the presence of asymptomatic carriers in the herd and the difference in disease progression should also be considered [36,37]. *Stenotrophomonas* was the dominant genus detected in normal lungs in our study. It is an emerging zoonotic bacterium that typically infects the respiratory tract [38]. *Stenotrophomonas* can be isolated from aqueous associated environment, including from soil, plant roots, lakes, and rivers, as well the wastewater from livestock farming [39]. In addition to its direct effects on the respiratory tract, resistance to commonly used antibiotics such as beta lactams, cephalosporins, aminoglycosides, and even carbapenem and colistin is a growing concern [40]. However, there are no reports indicating that the pathogen has a negative correlation or provides protection against any other respiratory pathogenic bacteria. *Stenotrophomonas* is not highly virulent, but it has emerged as an important part of opportunistic infection. It can become pathogenic under conditions of secondary infection or immunocompromising [38,39,41]. Because *Stenotrophomonas* is a zoonotic bacterium, the risk of transmission of both the pathogen and its antibiotic resistance genes to farm workers who have direct contact with animals is significant. Therefore, extra precautions (e.g., wearing masks and gloves, maintaining personal hygiene, and ensuring proper sanitation) should be taken during work, and regular health check-ups are recommended.

Considering the predominant pathways detected at higher abundance levels in the pneumonic samples, most are involved in amino acid, lipid, and cofactor metabolism. For example, the non-oxidative branch of the pentose phosphate pathway (NONOXIPENT-PWY) is linked to phenylpropanoid synthesis [42]. The Bifidobacterium shunt (P124-PWY) converts substrates into short-chain fatty acids (SCFAs) and other organic compounds. Additionally, pyruvate fermentation to isobutanol (PWY-7111) enhances isobutanol production by suppressing pyruvate dehydrogenase activity and activating NADPH regeneration in both the cytosol and mitochondria [43]. These biochemical reactions play a critical role in the uptake and utilization of inorganic and organic compounds necessary for growth and cellular homeostasis [44]. The greater abundance detected in the pneumonic group suggests increased metabolic activity within the microbial communities. It is highly likely that *Mycoplasma* spp., the main pathogenic bacteria identified in this herd, influenced the study’s results in this way. Furthermore, while PICRUSt2 is a widely used tool for predicting metabolic functions or pathways from 16S rRNA gene phylotypes, gene copy number is an important confounder in the inference of metabolic profiles [45]. Given that the average relative and absolute abundance of *Mycoplasma* spp. observed in the pneumonic group was higher than in the normal group, this could have impacted the predicted metabolic activity. However, validation of these pathways through experimental approaches, such as in vitro culture or animal model studies, is necessary to further substantiate these findings.

The lung is the best sample for representing the lower respiratory tract microbiomics in pigs, as it is constantly exposed to diverse microbial communities from inhaled air, the oropharynx, and other sources [17]. Consequently, lung tissue yields a higher DNA quantity compared to bronchoalveolar lavage (BAL) samples [46]. Additionally, BAL samples are more prone to contamination, particularly during the scalding step, where hot water can disrupt microbiome composition and introduce bacterial DNA from the skin of other carcasses [27].

Based on the facts in our study, the target farm is under a contract with unified management of the head office. It means that the pig health management, building facility, stocking density, staff training, biosecurity, and control requirements are standard. Despite all this, productive factors might be affected by the pathogen’s virulence, location, or surrounding environmental factors [22]. To solve this, additional protocols such as alternative vaccine programs or medication strategies should be implemented. Specifically, vaccination against *Mycoplasma hyopneumoniae* should be recommended as a first criterion, as it is the most isolated co-agent in multifactorial syndromes affecting the respiratory system in the modern pig industry [34]. Although the vaccine does not prevent infection, it provides partial protection that can reduce respiratory signs and the severity of lung lesions and aims to improve pig growth performance [7,47]. Macrolide and pleuromutilin are the most recommended antibiotics for the prevention and treatment of many bacteria causing respiratory problems. They should be administered for about 1–3 weeks, starting a week before the expected onset of the disease for control purposes. Notably, continuous medication during multiple production stages should be discouraged due to the development of antimicrobial resistance and increased risk of residues in pig carcasses [3,34]. However, the pigs that do not reach the standard market weight are usually recommended for being sent to slaughter to avoid further negative impacts such as re-infection or the increasing of production costs and to adhere to the all-in, all-out approach [7].

## 5. Conclusions

This is the first comprehensive study of pig lung microbiomics in Thailand. We prove that the slaughterhouse serves as a key checkpoint for assessing pig respiratory health, and the lung is representative of the lower respiratory tract for microbiome investigations. In our study, microbiome findings were correlated with lung phenotypes and pig growth performance. The detection of several pathogenic bacteria in both normal and pneumonic lungs suggests that nearly all pigs in the herd are at risk of developing respiratory disease, with some potentially serving as asymptomatic carriers. This can be explained by the multi-etiological nature of clinical respiratory disease. Pathogens can directly damage the respiratory tract, while intrinsic factors such as host susceptibility and exposure levels determine the likelihood of disease occurrence. Additionally, stressful conditions significantly contribute to disease severity and progression. Based on the acquired information, routine monitoring of lung lesions should be implemented across as many farms as possible to gain a more comprehensive understanding of the regional pig respiratory health status.

## Figures and Tables

**Table 1:** Description of gross lesions of 24 lungs tested in this study.

Sample ID	Category	Gross Description
A1	normal lung	Acute consolidation lesions affected 1% of the lung parenchyma
A2	normal lung	Acute consolidation lesions affected 1.5% of the lung parenchyma
A3	normal lung	Chronic consolidation lesions affected 0.5% of the lung parenchyma
A4	normal lung	Acute consolidation lesions affected 0.5% of the lung parenchyma
A5	normal lung	Acute consolidation lesions affected 1% of the lung parenchyma
A6	normal lung	No lesion
A7	normal lung	Acute consolidation lesions affected 0.5% of the lung parenchyma
A8	normal lung	Acute and chronic consolidation lesions affected 1% and 0.5% of the lung parenchyma
A9	normal lung	No lesion
A10	normal lung	No lesion
B1	pneumonic lung	Acute consolidation lesions affected 2% of the lung parenchyma with extensive pleuritis throughout the cranial and middle of the lung lobes
B2	pneumonic lung	Severe pleuritis and adhesion in the plural cavity cover more than 60–70% of the lung parenchyma with fibrino necrosis and hemorrhage on the cranial lobe
B3	pneumonic lung	Chronic consolidation lesions affected 10% of the lung parenchyma with severe pleuritis and adhesion in the plural cavity cover more than 60–70% of the lung parenchyma
B4	pneumonic lung	Acute consolidation lesions affected 11% of the lung parenchyma with mild pluritis at the caudal lobes
B5	pneumonic lung	Acute consolidation lesions affected 11% of the lung parenchyma
B6	pneumonic lung	Chronic consolidation lesions affected 15% of the lung parenchyma and diffusion of mottled areas
B7	pneumonic lung	Chronic consolidation lesions affected 15% of the lung parenchyma
B8	pneumonic lung	Chronic consolidation lesions affected 15% of the lung parenchyma
B9	pneumonic lung	Acute and chronic consolidation lesions affected 10% and 5% of the lung parenchyma, respectively
B10	pneumonic lung	Acute consolidation lesions affected 11% of the lung parenchyma
B11	pneumonic lung	Acute consolidation lesions affected 11% of the lung parenchyma
B12	pneumonic lung	Acute consolidation lesions affected 11% of the lung parenchyma
B13	pneumonic lung	Acute consolidation lesions affected 11% of the lung parenchyma
B14	pneumonic lung	Acute consolidation lesions affected 11% of the lung parenchyma

## Data Availability

No new data were created in this study.
